# Valorization of Sardinian Grapevine Leaves as a Sustainable Source of Flavonols with Anticancer Potential

**DOI:** 10.3390/antiox15091153

**Published:** 2026-09-10

**Authors:** Ylenia Spissu, Maria Lauda Tomasi, Carla Cossu, Andrea Floris, Emanuela Azara, Gavina Rita Serra, Irene Marchesi, Francesco Paolo Fiorentino, Gaia Rocchitta, Riccarda Zappino, Antonio Barberis

**Affiliations:** 1 Institute of Sciences of Food Production (ISPA), 07100 Sassari, Italy; gavina.serra@cnr.it; 2 Karsh Division of Gastroenterology and Hepatology, Department of Medicine, Cedars-Sinai Medical Center, Los Angeles, CA 90048, USA; marialauda.tomasi@cshs.org (M.L.T.); andreafloris1179@gmail.com (A.F.); 3 Nostra Signora della Mercede Unified Hospital, Via Ospedale 1, 08045 Lanusei, Italy; carla.cossu@aslogliastra.it; 4 Institute of Biomolecular Chemistry, National Research Council, Traversa la Crucca, 3, Loc. Baldinca, 07100 Sassari, Italy; emanuelagigliola.azara@cnr.it; 5 Kitos Biotech s.r.l.s., Porto Conte Richerche, S.P. 55 Porto Conte-Capo Caccia, Km 8.400 Loc. Tramariglio, 07041 Alghero, Italy; irenemarchesi@gmail.com (I.M.); fpfiorentino@gmail.com (F.P.F.); 6 Medicine, Surgery and Pharmacy Department, University of Sassari, Viale San Pietro 43/b, 07100 Sassari, Italy; grocchitta@uniss.it; 7 Department of Biomedical Sciences, University of Sassari, Viale San Pietro 43/b, 07100 Sassari, Italy; r.zappino@studenti.uniss.it

**Keywords:** grapevine leaf extracts, flavonol glycosides, LC–HRMS, redox activity, ROS modulation, *c-myb*, *bax/bcl2* ratio, mitochondrial apoptosis, sustainable by-products, colorectal carcinoma

## Abstract

Grapevine (*Vitis vinifera* L.) leaves are an abundant agricultural by-product and an underexploited source of bioactive polyphenols. This study characterized hydroalcoholic leaf extracts from autochthonous Sardinian cultivars and evaluated their effects in RKO human colorectal carcinoma cells. LC–HRMS revealed flavonol-rich profiles dominated by glycosylated derivatives of quercetin, kaempferol, and isorhamnetin, with marked cultivar-dependent differences and higher total flavonol abundance in Cannonau and Nieddera. Cyclic voltammetry showed distinct redox reactivity among extracts, with Vermentino and Martellada Bianca displaying the strongest electrochemical responses, consistent with their modulation of intracellular reactive oxygen species. In RKO cells, the extracts reduced metabolic activity in a dose-dependent manner, with IC50 values ranging from 100.3 to 251.8 µg/mL. At the molecular level, treatments modulated c-myb expression and shifted the bax/bcl2 balance toward a pro-apoptotic profile. These effects were supported by DAPI staining, showing chromatin condensation and nuclear fragmentation, and by moderate activation of caspase-3/7. Overall, Sardinian grapevine leaf extracts showed cultivar-specific phenolic composition, redox activity, and pro-apoptotic effects in an in vitro colorectal cancer model, supporting their potential valorization as sustainable sources of bioactive compounds. Further studies addressing bioavailability, metabolism, and in vivo effects are required to assess their physiological relevance and translational potential.

## 1. Introduction

Colorectal cancer (CRC) is one of the leading causes of cancer-related mortality worldwide, despite significant advances in early diagnosis and therapeutic strategies [1]. The multifactorial nature of CRC, involving dysregulated proliferation, altered redox homeostasis, mitochondrial dysfunction, and resistance to apoptosis, has prompted increasing interest in preventive and complementary approaches based on bioactive dietary compounds [2]. In this context, plant-derived polyphenols have attracted considerable attention due to their potential to modulate multiple cancer-related pathways, often with limited toxicity toward non-tumor cells [3,4].

Among polyphenol-rich botanical matrices, grapevine (*Vitis vinifera* L.) products represent a particularly relevant source of bioactive compounds. While grapes, wine, and winery by-products such as pomace and seeds have been extensively investigated, grapevine leaves remain comparatively underexplored, despite being produced in large amounts as an agricultural by-product [5]. Previous studies have demonstrated that grapevine leaves are especially rich in flavonols, including glycosylated derivatives of quercetin, kaempferol, and isorhamnetin, compounds known for their antioxidant, anti-inflammatory, and anticancer properties [6,7,8,9,10]. Recent studies have also demonstrated that leaves from red grapevine cultivars may accumulate anthocyanins, although generally at lower concentrations than flavonols. Cyanidin-3-O-glucoside and peonidin-3-O-glucoside are the predominant anthocyanins reported in pigmented grapevine leaves and contribute not only to red coloration but also to photoprotection, antioxidant defense, and adaptation to environmental stress. Consequently, anthocyanins may represent an additional class of bioactive phytochemicals potentially contributing to the biological properties of grapevine leaf extracts [11,12]. Thus, rather than representing a largely unexplored matrix, grapevine leaves constitute an increasingly recognized source of phytochemicals whose cultivar-specific composition and functional properties remain important areas of investigation. The valorization of grapevine leaves therefore aligns with current sustainability and circular economy goals, offering an opportunity to convert low-value biomass into high-value functional ingredients [13].

In this framework, the geographical origin of the grapevine represents an additional and often overlooked factor influencing polyphenolic composition and biological activity. Grapevines cultivated in Sardinia are exposed to distinctive Mediterranean pedoclimatic conditions, including high solar radiation, limited water availability, and marked thermal excursions. These environmental factors are known to modulate plant secondary metabolism and may influence the accumulation and structural diversification of phenolic compounds, particularly flavonols involved in photoprotection and oxidative stress defense [14,15,16,17,18,19]. Moreover, Sardinia hosts a rich heritage of autochthonous *Vitis vinifera* cultivars, which have evolved under long-term environmental selection and represent a valuable reservoir of genetic and metabolic diversity [20,21].

Flavonols have been reported to exert dose-dependent effects in cancer cells, displaying antioxidant activity at low concentrations while promoting cytostatic or pro-apoptotic responses at higher doses, particularly in cells characterized by elevated basal oxidative stress [3,4,22]. In colorectal cancer models, these effects have been linked to mitochondrial dysfunction, redox imbalance, cell cycle perturbation, and activation of the intrinsic apoptotic pathway [23,24,25,26]. However, the biological activity of complex plant extracts depends not only on total polyphenol content, but also on their qualitative composition, including the degree of glycosylation and methylation, which can influence cellular uptake, metabolic stability, and molecular targets [10,27]. Despite the growing literature on grapevine leaf phytochemistry, comparatively limited information is available on the cultivar-specific phenolic profiles and biological properties of leaves from Sardinian grapevine germplasm. Comparative studies linking the phytochemical signatures of Sardinian cultivars to their redox properties and anticancer-related cellular responses remain limited. This represents a relevant knowledge gap, since genotype–environment interactions may generate distinctive phytochemical profiles with potential implications for both biological activity and the valorization of region-specific viticultural by-products.

Dietary flavonols generally exhibit limited systemic bioavailability, with their intestinal absorption and metabolism being strongly influenced by their glycosylation pattern. While some flavonol glycosides can be hydrolyzed and absorbed in the small intestine, a substantial fraction of unabsorbed compounds reaches the colon, where they undergo extensive metabolism by the gut microbiota, generating phenolic metabolites that may retain biological activity [28,29,30,31]. This metabolic fate provides a rationale for investigating the biological effects of grapevine leaf extracts in colorectal cellular models [28,29,30,31].

At the molecular level, transcription factors regulating proliferation and survival play a central role in CRC progression. c-MYB is increasingly recognized as a key regulator of colorectal tumor cell growth, cell cycle progression, and stress adaptation [32,33]. Altered MYB signaling has been associated with aggressive tumor behavior and resistance to therapy, while its suppression has been linked to reduced proliferative capacity and increased sensitivity to metabolic and apoptotic stress [34,35]. In parallel, the balance between pro- and anti-apoptotic members of the BCL-2 family, commonly expressed as the BAX/BCL2 ratio, represents a critical checkpoint in mitochondrial apoptosis and a frequent target of polyphenol-mediated anticancer activity [36,37].

Previous work from our research group characterized the phenolic profile of Vermentino grapevine leaves and demonstrated biological effects of the corresponding hydroalcoholic extract in breast cancer cell models, identifying FASN/SUMOylation-related mechanisms as potential molecular targets [38]. However, that study was restricted to a single cultivar and a different tumor model and did not address cultivar-dependent relationships among phytochemical composition, electrochemical redox behavior, and biological responses in colorectal cancer cells. Although the phenolic composition and antioxidant properties of grapevine leaves are now relatively well documented, important gaps remain regarding the extent to which cultivar-specific phytochemical differences translate into distinct redox and biological responses. This question is particularly relevant for autochthonous cultivars from geographically and environmentally distinctive viticultural regions such as Sardinia, for which comparative information remains limited. Furthermore, studies integrating detailed phytochemical profiling with electrochemical assessment of redox behavior and mechanistic evaluation of anticancer-related responses in colorectal cancer cells remain comparatively scarce. Establishing such composition–redox–bioactivity relationships may help move beyond the general recognition of grapevine leaves as a source of polyphenols toward the identification of cultivar-specific functional signatures relevant to their targeted valorization.

The present study aimed to (i) characterize and compare the phenolic profiles, including flavonols and anthocyanins, of hydroalcoholic leaf extracts from four *Vitis vinifera* cultivars traditionally grown in Sardinia; (ii) investigate cultivar-dependent differences in their electrochemical redox properties and evaluate their effects on cell viability, metabolic activity, and intracellular oxidative status in RKO human colorectal carcinoma cells; and (iii) investigate molecular pathways related to proliferation and apoptosis, with particular focus on *c-myb* expression, the *bax/bcl2* transcriptional balance, apoptotic nuclear morphology, and caspase-3/7 activation. By integrating chemical, electrochemical, functional, and molecular data, this study provides new insights into the biological activity of Sardinian grapevine leaf extracts and their potential valorization as sustainable, territorially distinctive sources of bioactive polyphenols.

## 2. Materials and Methods

### 2.1. Reagents and Solutions

Ethanol, 2-propanol BioReagent, chloroform, thiazolyl blue tetrazolium bromide powder (MTT), 200 mM glutamine, and penicillin–streptomycin solution were purchased from Merck Life Science (Milan, Italy). The phosphate-buffered saline (PBS) solution was prepared using NaCl (137 mM), KCl (2.7 mM), Na_2_HPO_4_ (8.1 mM) and KH_2_PO_4_ (1.47 mM) from Merck Life Science, and was adjusted to pH 7.4. Qubit RNA assay kits were obtained from Thermo Fisher Scientific (Waltham, MA USA). Eagle’s Minimum Essential Medium (EMEM), fetal bovine serum (FBS), MEM non-essential amino acid 100× and trypsin 0.25% EDTA solution were purchased from Euroclone (Milano, Italy).

### 2.2. Plants and Polyphenol Extraction

Mature leaves of two white (Vermentino and Martellada Bianca) and two red (Nieddera and Cannonau) *Vitis vinifera* L. cultivars were collected in August from the apical portion of plants grown in the collection field of the “Complesso Forestale di Pantaleo” in Santadi (Sulcis-Iglesiente), Italy (Latitude: 39.090199° N and Longitude: 8.797491° E, datum WGS84). As previously described [38], leaves were ground into a fine powder in liquid nitrogen to prevent the degradation of thermolabile compounds and then processed for polyphenol extraction.

Accelerated solvent extraction (ASE) was performed according to previously established methods [38,39], with some modifications, using a Dionex ASE 350 (Thermo Fisher Scientific Inc., Waltham, MA, USA) under the operating conditions reported in Table 1.

Powdered leaves (2 g) were weighed into a 22 mL stainless-steel extraction cell, and a hydroalcoholic ethanol/water (40:60, *v*/*v*) mixture was used as the extraction solvent. Three independent extractions were performed for each cultivar. After extraction, the hydroalcoholic extracts containing the polyphenolic fraction were frozen at −80 °C. Ethanol was removed under a nitrogen stream, and the samples were subsequently lyophilized to eliminate residual water and solvent traces. The resulting ethanol-free, freeze-dried extracts were used for chemical analyses and cell treatments. Extraction yield was calculated as the ratio between the weight of the freeze-dried extract recovered and the initial weight of leaf powder used and was expressed as mean yield (%) ± SD [39].

### 2.3. Chemical Characterization of Polyphenolic Extracts

The chemical characterization of Vermentino leaf extract was previously reported by our group [38]; therefore, the characterization described below focuses on the other three cultivars.

#### 2.3.1. LC-MS/DAD Analysis

Extracts from Martellada Bianca, Nieddera and Cannonau were filtered through 0.2 µm regenerated cellulose syringe filters (Phenomenex, Torrance, CA, USA). Phenolic compounds were determined by liquid chromatography–mass spectrometry (LC–MS), according to previously described methods [38,40]. A diode array detector (DAD) was used at 280, 320, and 520 nm for quantitative analysis. Quantification of phenolic compounds was performed using external calibration curves generated on commercial standards. The content of quercetin 3-O-(6-acetyl)-glucoside was estimated using a quercetin 3-O-glucoside standard curve.

#### 2.3.2. LC-HRMS Analysis

As previously reported [38], high resolution mass spectrometry (HRMS) analyses were performed by a Q Exactive Orbitrap (Thermo Scientific, Bremen, Germany) coupled to an Agilent 1200 Series HPLC system (Agilent Technologies, Santa Clara, CA, USA), equipped with a binary pump, thermostatted autosampler, and column oven set at 39 °C. To investigate the secondary metabolite profile, the Q Exactive system was equipped with a heated electrospray ionization (HESI) source operating in both positive and negative ion modes. The HESI parameters were as follows: spray voltage, 3.2 kV; sheath gas flow rate, 35 arbitrary units; auxiliary gas flow rate, 10 arbitrary units; sweep gas flow rate, 2 arbitrary units; and capillary temperature, 300 °C. Full MS acquisition was performed at a resolution of 70,000 full width at half maximum (FWHM) for precursor ions and 17,500 for fragment ions, with mass accuracy of 5 ppm. The MS parameters were as follows: automatic gain control (AGC) target 1 × 10^6^; maximum injection time (IT), 200 ms; and scan range, 100–1200 *m*/*z*. Xcalibur 3.1.66 software (Thermo Scientific, Bremen, Germany) was used for instrument control and data processing. Chromatographic separation was carried out using a Gemini C18 (Phenomenex, Torrance, CA, USA; 100 × 2.1 mm, 3 µm, 100 Å). The flow rate was 0.2 mL/min over a 55 min run, with an injection volume of 5 µL. A linear gradient elution was applied using solvent A (0.2% acetic acid in water) and solvent B (acetonitrile) as follows: 0 min, 10% B; 0–20 min, 10–20% B; 20–40 min, 20–40% B; and 40–50 min, 40–70% B. The column was equilibrated for 8 min before each analysis. Compounds were identified based on retention time relative to external standards (tR), UV-Vis spectra (200–650 nm), high resolution mass spectra, phytochemical libraries, and reference literature. Quantification of individual phenolic compounds was performed using calibration curves of the corresponding reference standards. When reference standards were not available, quantification was based on structurally related compounds.

### 2.4. Electrochemical Characterization by Cyclic Voltammetry

The redox characteristics of *Vitis vinifera* leaf extracts were assessed by cyclic voltammetry (CV) to investigate their electron-transfer properties and overall redox behavior. The experimental procedure was adapted from previously published methods with minor modifications [41,42]. Measurements were carried out using disposable screen-printed electrodes (GSI Technologies, Burr Ridge, IL, USA), consisting of a 5 mm carbon working electrode (WE), a Ag/AgCl pseudo-reference electrode (RE), and a carbon counter electrode (AE). Electrochemical signals were recorded using a Quadstat four-channel potentiostat coupled with an e-Corder 410 acquisition unit and Echem software v2.1.0 (eDAQ Europe, Warsaw, Poland).

Voltammograms were acquired over a potential window ranging from −0.2 V to +0.8 V (vs. Ag/AgCl pseudo-reference electrode) at a scan rate of 0.1 V s^−1^. Prior to sample analysis, a baseline was established by depositing 70 µL of phosphate-buffered saline (PBS) used as supporting electrolyte, onto the working electrode surface. After recording the background signal, the electrolyte was gently removed using absorbent paper, taking care not to damage the electrode surface. Subsequently, 70 µL of grapevine leaf extract (2 mg/mL) was deposited onto the electrode to obtain the corresponding voltammetric response. All measurements were performed in triplicate.

For comparative purposes, the voltammograms were integrated, and the area under the curve (AUC) was calculated up to +0.5 V, with results expressed in microcoulombs (µC). As reported in previous studies [43], this potential can be considered a threshold for evaluating the redox activity of phenolic compounds. Signals recorded at potentials above +0.5 V are generally associated with compounds exhibiting lower reducing power and were therefore not considered major contributors to antioxidant activity in this study.

The resulting AUC values were used to compare the electrochemical profiles of the different grapevine leaf extracts, providing a quantitative measure of their relative redox reactivity.

### 2.5. Cell Viability Assay

RKO human colon carcinoma cells were obtained from ATCC (Accession number: ATCC CRL-2577; LCG Standards, Milan, Italy). Cells were maintained in EMEM supplemented with 10% FBS, 1% penicillin/streptomycin, 2 mM glutamine and 1× MEM non-essential amino acids at 37 °C in a humidified atmosphere (95%) containing 5% CO_2_.

RKO cells were plated in 96-well plates (10 × 10^4^/100 µL) and exposed to increasing concentrations of leaf EtOH/H_2_O extracts (50, 100, 250, 500 μg/mL) for 24 h. The concentration range (50–500 µg/mL, expressed as dry extract weight) was selected to encompass low, intermediate, and high exposure levels and to characterize the concentration-dependent effects of the extracts on RKO cell metabolic activity. After 24 h of treatment, cells were incubated with 100 µL (0.5 mg/mL) of MTT at 37 °C for 3 h. After incubation, the MTT solution was removed, and the resulting formazan crystals were dissolved in 100 µL of 2-propanol. Absorbance was measured at 570 nm using a microplate reader (Sunrise™ Absorbance Reader, Tecan Trading AG, Männedorf, Switzerland). Cell viability was expressed as a percentage relative to untreated control cells. The viability data were obtained from three independent experiments.

### 2.6. Reactive Oxygen Species (ROS) Measurement

The intracellular levels of hydrogen peroxide (H_2_O_2_) were measured using the ROS-Glo H_2_O_2_ assay (Promega G8820, Milano (MI), Italy), according to the manufacturer’s instructions. RKO cells were trypsinized and seeded in white 384-well plates at a density of 2000 cells per well in 20 μL of fresh medium. Cells were suspended in MEM supplemented with 10% FBS, 2 mM L-glutamine and 1× non-essential amino acids.

After 22 h, 5 μL of 5× concentrated Vermentino and Martellada Bianca extracts (50, 100, 250 and 500 μg/mL), suspended in MEM without supplements, were added to each well. All samples, including untreated controls, contained 0.17% DMSO except for Menadione-treated samples which contained 0.1% DMSO and were used as a positive control for ROS generation. Menadione docks into the active site of NAD(P)H Quinone Dehydrogenase 1 (NQO1) enzyme, enabling its own reduction (PMID: 17088248). Back-oxidation of Menadione generates ROS (O^2−^) when molecular oxygen is present (redox cycling, PMID: 2820459).

Immediately after treatment, 5 µL of H_2_O_2_ substrate was added to each well. After 3 h of incubation, 30 µL of freshly prepared detection reagent was added. Following 20 min of incubation at room temperature, luminescence was measured using a GloMax^®^ Discover microplate reader (Promega, Milano (MI), Italy).

All conditions were tested in three technical replicates. All liquid handling steps were performed using an automated pipetting platform (Gilson PIPETMAX^®^, Merck Life Science, Milan, Italy).

### 2.7. Quantitative RT-PCR

Total RNA was extracted from RKO using EUROGOLD Trifast reagent (Euroclone, Milan, Italy) and 500 ng of RNA was reverse-transcribed using the LunaScript^®^ RT SuperMix Kit (New England Biolabs, Ipswich, MA, USA) under the following thermal conditions: 25 °C for 2 min, 55 °C for 10 min and 95 °C for 1 min. cDNA was subsequently subjected to quantitative RT-PCR using SYBR Green chemistry to assess the expression of *c-myb*, *bax*, *bcl2*, and the housekeeping gene 18S (Qiagen, Milan, Italy). The thermal profile consisted of an initial denaturation step at 95 °C for 1 min, followed by 40 cycles at 95 °C for 15 s and at 60 °C for 30 s. The cycle threshold (Ct) values of target genes were normalized to the Ct value of 18S rRNA to obtain ΔCt values. Relative expression of target genes was calculated according to the formula 2^−ΔΔCt^, where ΔΔCt = ΔCt of target genes in experimental conditions−ΔCt of target gene under control conditions [44]. The mRNA data were obtained from three independent experiments.

### 2.8. DAPI Staining

RKO cells (10 × 10^5^) were seeded in 6-well plates and, after 24 h, treated with increasing concentrations of grapevine leaf EtOH/H_2_O extracts for 24 h. Cells were then fixed with 4% paraformaldehyde at room temperature (RT) for 15 min and washed twice with PBS. To detect apoptotic nuclear morphology, cells were stained with DAPI at a final concentration of 300 nM and incubated at RT for 2 h in the dark. After staining, cells were washed three times with PBS, and fluorescence was analyzed using a fluorescence microscope (EVOS Cell Imaging System, Thermo Fisher Scientific). Data were obtained from three independent experiments.

### 2.9. Apoptosis Assay

Caspase-Glo 3/7 assay (Promega G8091, Milano (MI), Italy) was used to measure the level of caspase activity in cultured cells, according to the manufacturer’s instructions. RKO cells were trypsinized and plated in white 384-well plates (Corning 3570, Merck Life Science, Milan, Italy) at a density of 5 × 10^3^ cells per well in 20 μL of fresh medium. Cells were suspended in complete medium without phenol red. After 24 h, Etoposide 4 μM or Doxorubicin 0.25 μM and natural extracts (10, 50 or 100 μg/mL) were added to each sample, suspended in complete medium without phenol red, as single agents. All samples, including untreated cells, contained DMSO 0.16%. After 24 h of treatment, 25 μL of freshly prepared Caspase-Glo reagent was added to each sample. After 1 h of incubation, luminescence was measured with GloMax Discover Microplate Reader (Promega). Three technical replicates were performed for each condition. All the steps described were performed using Gilson Pipetmax (Merck Life Science, Milan, Italy).

### 2.10. Statistical Analysis

Statistical analysis was performed using GraphPad Prism 8 for Windows software (GraphPad Software, Inc., La Jolla, CA, USA). Data were presented as mean ± standard deviation (SD) in the figures. Data were analyzed by one-way ANOVA followed by Dunnett’s multiple comparisons test to compare each treatment condition with the corresponding untreated control. Statistical significance was set at *p* ≤ 0.05 and *p* ≤ 0.01.

## 3. Results and Discussion

### 3.1. Characterization of Vitis vinifera Leaf Extracts

LC–HRMS analysis of hydroalcoholic extracts obtained from *Vitis vinifera* leaves (cv. Martellada Bianca, Nieddera, and Cannonau) (Table 2, Figure S1) revealed a phenolic profile dominated by glycosylated flavonols, mainly derivatives of isorhamnetin, quercetin, and kaempferol, with minor contributions from myricetin 3-O-glucoside and eriodictyol 7-O-glucoside. This compositional pattern is consistent with previous reports on grapevine leaves, which identify flavonol glycosides as the predominant phenolic class, typically exceeding phenolic acids and stilbenes in abundance [6,7,8,9,13].

Quantitatively, Cannonau and Nieddera extracts displayed higher total flavonol content than Martellada Bianca. In particular, isorhamnetin glucoside was the major constituent in both Cannonau and Nieddera (22.32 ± 0.07 mg g^−1^ and 14.67 ± 0.03 mg g^−1^ respectively), and accounted for a large fraction of the total quantified compounds (85.75% in Cannonau and 61.98% in Nieddera), whereas Martellada Bianca exhibited a more balanced distribution among isorhamnetin (49.4%), quercetin (29.68%), and kaempferol derivatives (19.63%). Elevated levels of isorhamnetin glycosides have previously been described in Mediterranean grapevine cultivars and may reflect cultivar-dependent differences in O-methylation reactions within the flavonol biosynthetic pathway [5,7].

Quercetin 3-O-glucoside was detected in all cultivars and was particularly abundant in Nieddera (5.17 ± 0.05 mg g^−1^ corresponding to 21.85% of total polyphenols). This finding is consistent with the phenolic profile reported for *V. vinifera* cv. Vermentino leaves, in which quercetin 3-O-glucoside and isorhamnetin glucoside represent the two dominant compounds in hydroalcoholic extracts [38]. However, while Vermentino exhibits a relatively balanced contribution of these two flavonols, Cannonau shows a marked predominance of the methylated derivative, suggesting cultivar-specific differences in flavonols tailoring reactions. Comparable trends have been reported in other studies comparing grapevine leaves and related matrices, where quercetin and kaempferol 3-O-glucosides are consistently identified as key components, with variable proportions depending on genotype and environmental conditions [13,45,46].

The distribution of kaempferol derivatives further discriminated the cultivars. Martellada Bianca showed a relatively high contribution of kaempferol 3-O-glucoside together with detectable galactoside and rutinoside forms, resulting in a more diversified flavonol profile. In contrast, Cannonau was nearly devoid of kaempferol galactosides and rutinosides, reinforcing the hypothesis of a metabolic specialization toward isorhamnetin accumulation. Similar cultivar-dependent variability in kaempferol versus quercetin derivatives has been documented in grapevine leaves and has been proposed as a useful chemotaxonomic and functional marker [6,7,8].

Quercetin 3-O-(6-acetyl)-glucoside was detected in Martellada Bianca but not in Nieddera or Cannonau. This compound has also been reported in Vermentino leaves [38] and in studies on grapevine foliage [47,48], but it is not ubiquitously present across cultivars, indicating cultivar-dependent differences in the occurrence of acetylated flavonol derivatives.

Besides flavonols, targeted LC-MS analysis revealed the presence of anthocyanins in the leaf extracts of the red cultivars. As shown in Table 3 and in Figure S2B in Supplementary Materials, Nieddera displayed a markedly richer anthocyanin composition than Cannonau. Cyanidin-3-O-glucoside was the predominant anthocyanin detected, followed by peonidin-3-O-glucoside, whereas petunidin-, malvidin-, and coumaroylated derivatives were detected in lower amounts. In contrast, Cannonau showed a much lower anthocyanin content, with only cyanidin-3-O-glucoside and peonidin-3-O-glucoside detected at quantifiable levels, while the remaining anthocyanins were below the limit of quantification.

Although flavonols represented the predominant phenolic class in all extracts, the identification of anthocyanins in Nieddera provides additional insight into the phytochemical complexity of this cultivar (Table 3). Cyanidin-3-O-glucoside and peonidin-3-O-glucoside were the major anthocyanins detected, in agreement with previous reports describing these pigments as the principal anthocyanins accumulated in pigmented grapevine leaves [12]. Anthocyanins have long been recognized as multifunctional metabolites involved in photoprotection, scavenging of reactive oxygen species, and adaptation to environmental stresses such as intense solar radiation, drought, and ultraviolet exposure. Their biosynthesis is tightly associated with the activation of the phenylpropanoid pathway and environmental regulation of secondary metabolism [11]. Although their quantitative contribution was considerably lower than that of flavonols, anthocyanins may contribute to the overall redox properties of Nieddera leaf extract. However, the relative contribution of individual phenolic classes and possible interactions among extract constituents cannot be established from the present data.

Overall, although the three cultivars share a conserved “core” flavonol fingerprint typical of grapevine leaves, they display distinct cultivar-specific metabolic signatures. Cannonau appears highly enriched in isorhamnetin glucoside, Nieddera shows a strong contribution of quercetin 3-O-glucoside, and Martellada Bianca is characterized by greater structural diversity, including acetylated flavonol derivatives. This variability is fully consistent with the literature on grapevine leaf polyphenols and highlights the relevance of cultivar selection when considering leaves as a source of bioactive compounds [5,13].

### 3.2. Redox Activity of Vitis vinifera Extracts

**Figure 1:**
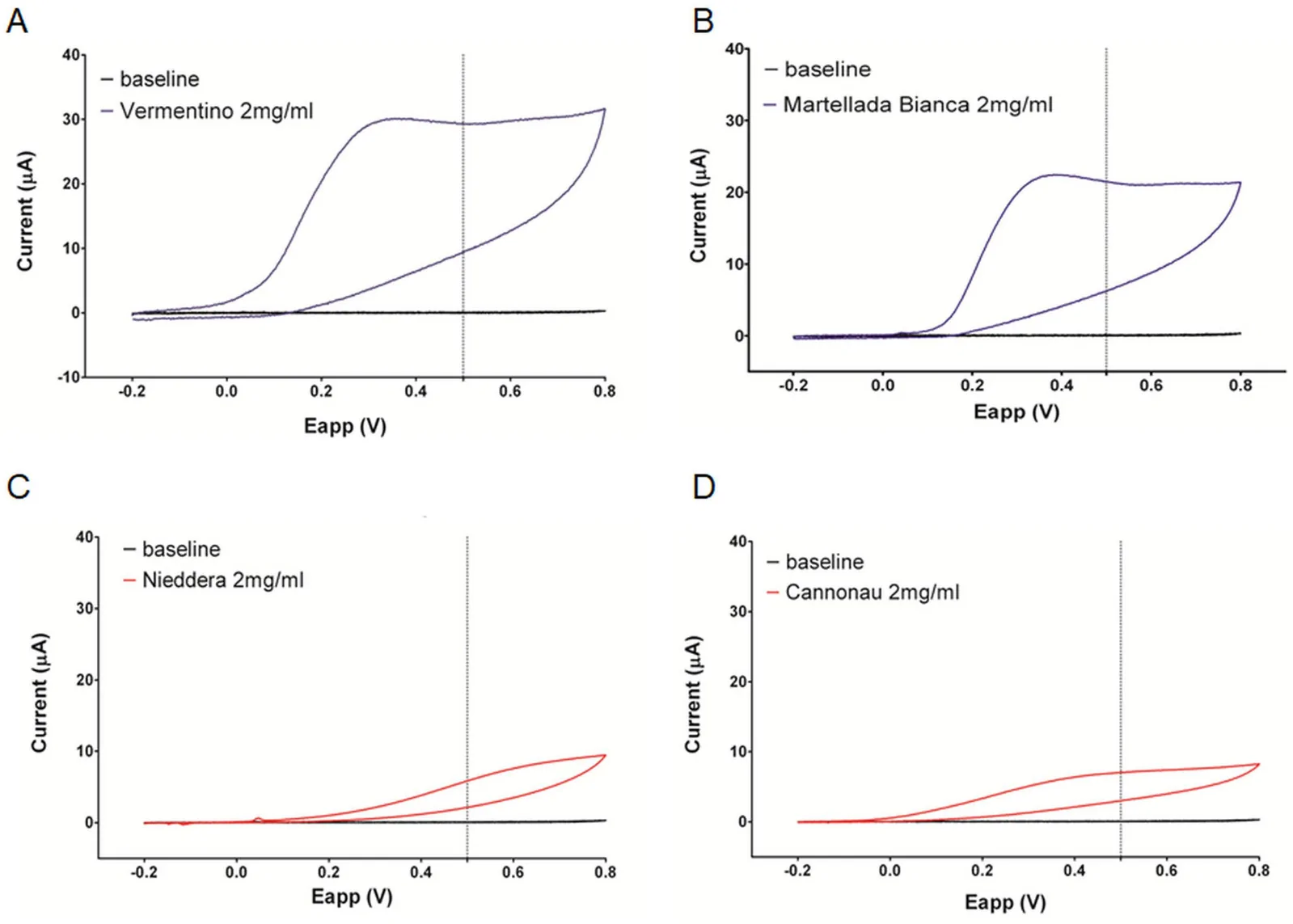
Cyclic voltammograms of hydroalcoholic extracts obtained from *Vitis vinifera* leaves (blue and red lines, black line is the baseline): (**A**) Vermentino, (**B**) Martellada Bianca, (**C**) Nieddera, and (**D**) Cannonau. Measurements were performed in PBS using screen-printed carbon electrodes over a potential range of −0.2 to +0.8 V at a scan rate of 0.1 V s^−1^.

Cyclic voltammetry analysis revealed clear cultivar-dependent differences in the electrochemical behavior of the grapevine leaf extracts (Figure 1).

All extracts displayed oxidation waves that were absent in the baseline electrolyte, confirming the presence of electroactive phenolic species.

Among the investigated cultivars, Vermentino (Figure 1A) exhibited the most pronounced voltammetric response, with a steep increase in anodic current starting at approximately 0.2–0.3 V and reaching the highest anodic currents within the scanned potential range. Integration of the voltammetric profiles up to +0.5 V confirmed this trend, yielding an AUC value of 10.17 µC and indicating the highest electrochemical activity among the analyzed extracts. Martellada Bianca (Figure 1B) also showed a strong electrochemical response, although lower than that of Vermentino, with an AUC value of 6.16 µC. In contrast, Cannonau (Figure 1D) exhibited a moderate voltammetric response (AUC = 2.01 µC), whereas Nieddera (Figure 1C) displayed the lowest AUC value (0.998 µC).

The redox activity of the grapevine leaf extracts therefore followed the order Vermentino >> Martellada Bianca > Cannonau > Nieddera. This ranking did not parallel the total flavonol content determined by LC–HRMS, since Cannonau showed the highest total flavonol concentration but a substantially lower AUC than Vermentino. This apparent discrepancy reflects the different information provided by the two analytical approaches. Whereas LC–HRMS quantifies the abundance of the identified flavonols, the voltammetric AUC calculated up to +0.5 V preferentially reflects the contribution of readily oxidizable compounds within this potential range. Indeed, the contribution of individual phenolic compounds to AUC depends not only on their concentration but also on their oxidation potential and structural characteristics. Consequently, highly abundant compounds oxidized at relatively high potentials may contribute less to AUC0.5 than less abundant but more readily oxidizable compounds, as previously demonstrated in complex grape-derived matrices [43]. Therefore, the higher AUC observed for Vermentino is consistent with a greater contribution of low-potential electroactive constituents and should not be interpreted simply on the basis of total flavonol abundance.

**Figure 2:**
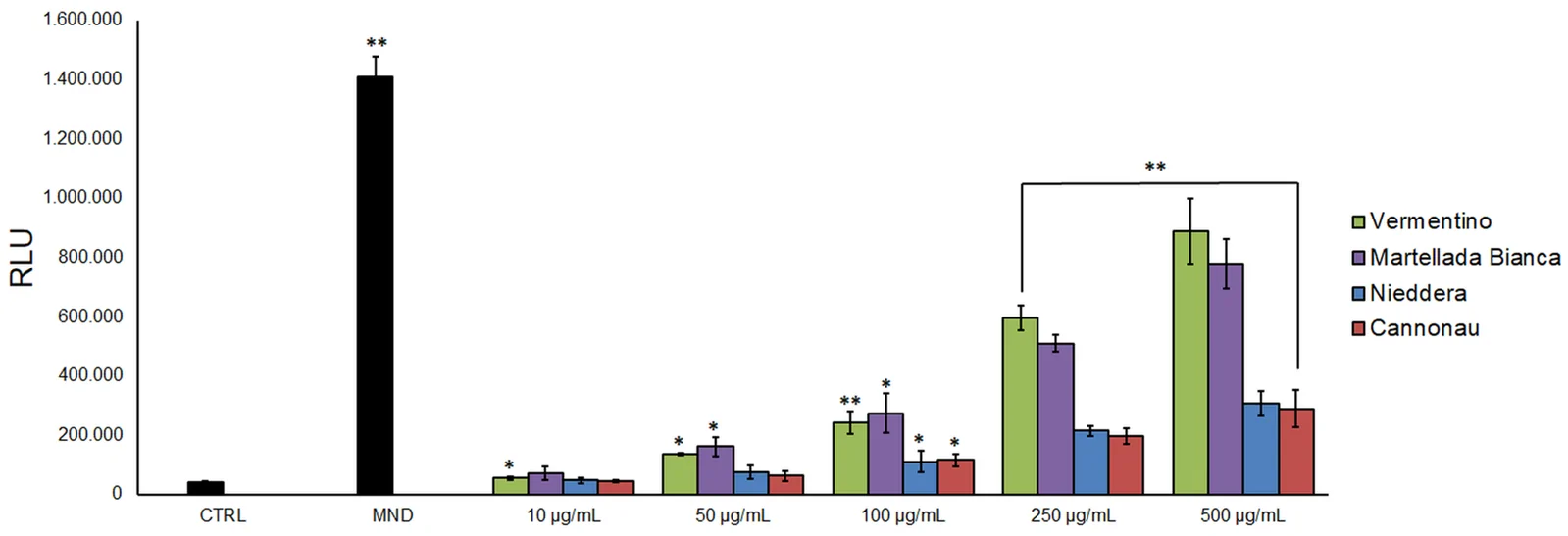
Intracellular ROS levels in RKO cells treated with grapevine leaf extracts. Cells were exposed to increasing concentrations of extracts, and H_2_O_2_ production was quantified using the ROS-Glo™ assay. Data are presented as mean ± SD. Statistical significance was assessed by one-way ANOVA followed by Dunnett’s multiple comparisons test. *p* ≤ 0.05 was considered statistically significant (* *p* ≤ 0.05; ** *p* ≤ 0.01).

The higher electrochemical activity detected for Vermentino and Martellada Bianca was accompanied by a marked modulation of intracellular ROS levels in RKO cells treated with these extracts (Figure 2). Although these observations are consistent with the redox-active nature of the extracts, a direct quantitative relationship between voltammetric AUC and intracellular ROS modulation cannot be established from the present data.

Taken together, the cyclic voltammetry results highlight cultivar-dependent differences in the redox behavior of grapevine leaf extracts and indicate that electrochemical activity cannot be predicted from total flavonol abundance alone. Rather, chromatographic composition and electrochemical behavior provide complementary information for the characterization of the redox properties of these complex phytochemical matrices.

### 3.3. Effects of Grapevine Leaf Extracts on Cells Viability and c-myb Downregulation

The hydroalcoholic extracts obtained from Vitis vinifera leaves exhibited a flavonol-rich profile dominated by glycosylated derivatives of isorhamnetin, quercetin, and kaempferol, with cultivar-dependent quantitative differences. This compositional fingerprint is consistent with previous reports on grapevine leaves and related matrices, which identify flavonols as major contributors to antioxidant and biological activity [3,4,10,49,50]. Extracts enriched in methylated flavonols, such as isorhamnetin glycosides, have been associated with enhanced metabolic stability and cellular bioactivity.

**Figure 3:**
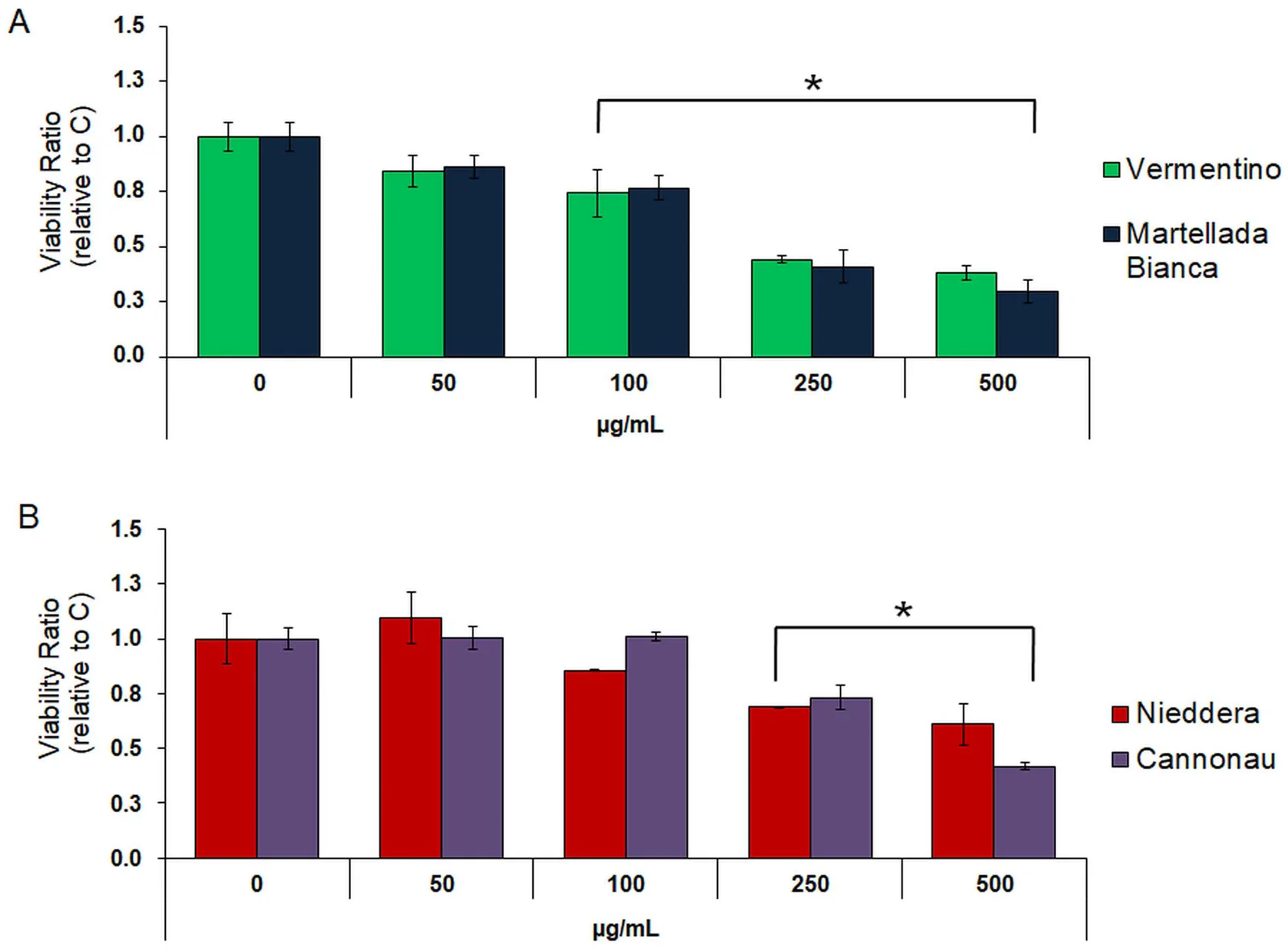
Effects of *Vitis vinifera* leaf extracts on RKO cell viability assessed by the MTT assay. Cells were treated with increasing concentrations (50–500 µg/mL) of Vermentino and Martellada Bianca (**A**), and with Nieddera and Cannonau extracts (**B**) for 24 h. Cell viability is expressed as a percentage relative to untreated control cells. Data are presented as mean ± SD. Statistical significance was assessed by one-way ANOVA followed by Dunnett’s multiple comparisons test. * *p* ≤ 0.05 was considered statistically significant.

The biological relevance of these compositional differences was evaluated in RKO human colorectal carcinoma cells using the MTT assay (Figure 3).

As shown in Figure 3, all leaf extracts induced a dose-dependent reduction in cell viability, with no significant effects at low concentrations and statistically significant decreases at intermediate and high doses. The calculated IC_50_ values were 127.0 µg/mL (95% CI: 14.8–230.1) for Vermentino, 150.3 µg/mL (95% CI: 22.57–278.1) for Martellada Bianca, 251.8 µg/mL (95% CI: 0.0–389.9) for Nieddera, and 147.8 µg/mL (95% CI: 53.7–327.4) for Cannonau. This pattern is characteristic of polyphenol-rich mixtures in colorectal cancer models and reflects a transition from neutral or antioxidant effects (minimal or antioxidant-like effects) at low doses to cytostatic or cytotoxic responses at higher concentrations [3,4,51]. Importantly, some extracts characterized by higher total flavonol content and enrichment in isorhamnetin and quercetin derivatives showed pronounced reductions in MTT signal. Although this pattern suggests a possible association between phytochemical composition and biological activity, it does not establish a causal contribution of these individual compounds or phenolic classes.

Because MTT reduction is closely linked to cellular reductive metabolism and mitochondrial dehydrogenase activity, the observed decrease in MTT signal suggests an alteration in cellular metabolic competence. Although mitochondrial function was not directly evaluated in the present study, these findings are consistent with previous reports showing that flavonols can affect redox homeostasis and mitochondrial-associated metabolic pathways in colorectal cancer cells. RKO cells exhibit elevated basal oxidative stress and strong dependence on mitochondrial metabolism, rendering them especially sensitive to ROS-mediated perturbations [24,25,26,52]. Under these conditions, flavonol-induced ROS accumulation may contribute to metabolic stress and alterations in cellular redox balance, which could ultimately affect mitochondrial function and energy metabolism, all of which contribute to diminished MTT reduction. The modulation of antioxidant enzymes may also be influenced by the presence of anthocyanins, which have been shown to regulate cellular antioxidant defenses through ROS-dependent signaling mechanisms [53]. Although their concentration was considerably lower than that of flavonols, these compounds could contribute to maintaining intracellular redox balance in combination with flavonol glycosides [54].

**Figure 4:**
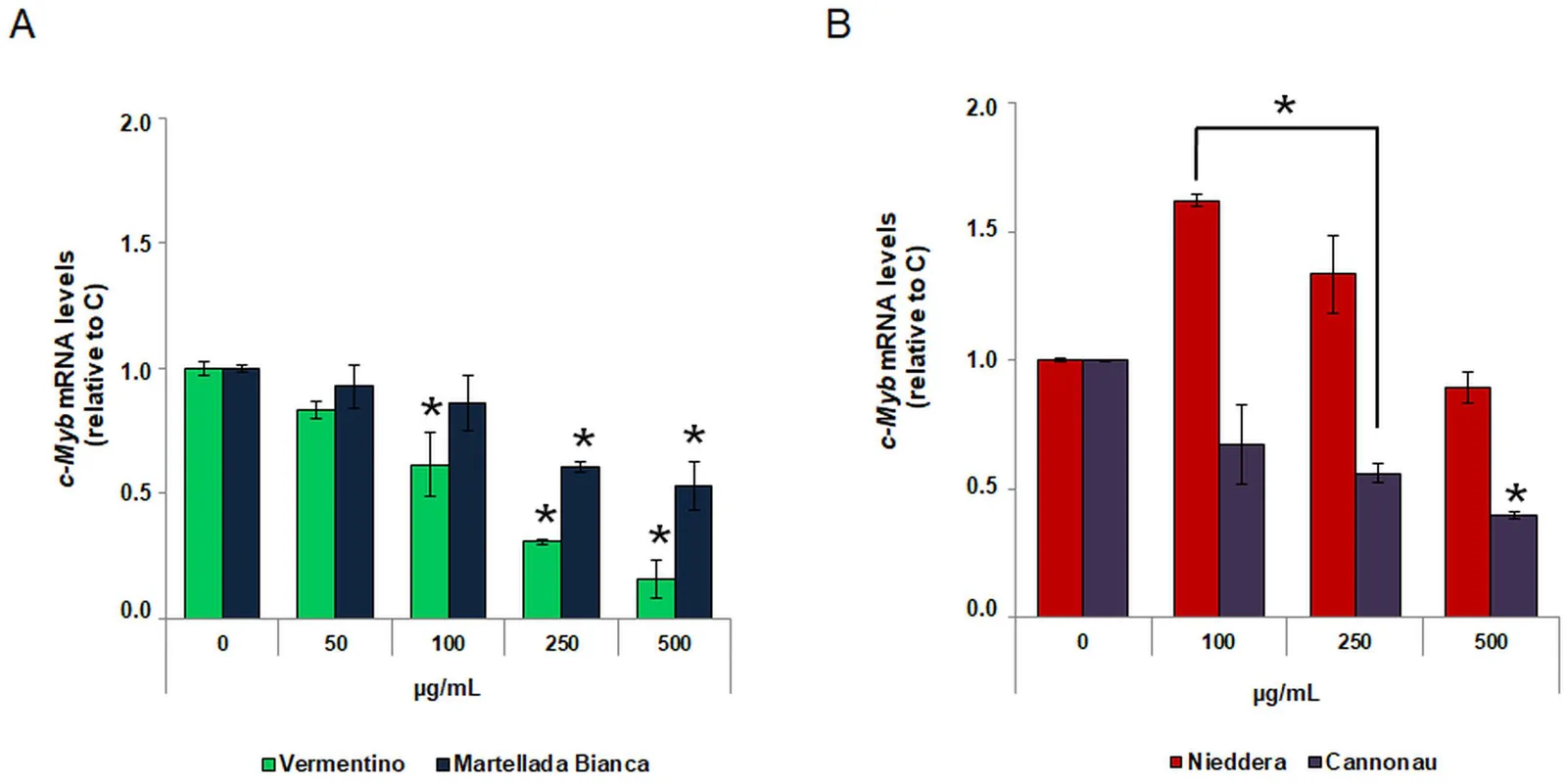
(**A**,**B**) Relative expression of *c-myb* mRNA in RKO cells treated with *Vitis vinifera* leaf extracts. Cells were exposed to increasing concentrations of extracts for 24 h, and gene expression was analyzed by qRT-PCR using the 2^−ΔΔCt^ method, normalized to 18S rRNA. Data are presented as mean ± SD. Statistical significance was assessed by one-way ANOVA followed by Dunnett’s multiple comparisons test. * *p* ≤ 0.05 was considered statistically significant.

To further explore the molecular basis of the observed cytostatic/cytotoxic effects, the expression of *c-myb* mRNA was analyzed (Figure 4).

*c-myb* expression displayed a dose- and condition-dependent modulation. Treatment with Vermentino, Martellada Bianca, and Cannonau extracts resulted in progressive downregulation, whereas Nieddera induced a biphasic response, characterized by transient upregulation at intermediate concentrations followed by reduced expression at the highest dose (500 µg/mL). Notably, the strongest reductions in cell viability coincided with conditions showing decreased c-MYB expression, suggesting a functional link between metabolic impairment and repression of proliferation-associated transcriptional programs. This association is biologically plausible, as MYB is a key transcription factor regulating cell cycle progression, proliferation, and survival in colorectal cancer. Its overexpression has been linked to tumor growth and metastatic behavior, whereas its suppression is associated with reduced proliferative capacity and increased sensitivity to metabolic and oxidative stress [32,33]. Flavonols such as quercetin and isorhamnetin derivatives have been reported to modulate redox-sensitive signaling pathways involved in cell proliferation, apoptosis, and cell-cycle regulation. Recent evidence suggests that anthocyanins, particularly cyanidin derivatives, can interfere with transcriptional networks regulating proliferation and oxidative stress responses. Therefore, the anthocyanin fraction detected in Nieddera may complement the activity exerted by flavonols on c-MYB-dependent signaling pathways. Since c-MYB is a key transcriptional regulator of colorectal cancer growth and progression, the reduction in *c-myb* expression observed in treated cells may contribute to the antiproliferative effects of grape leaf extracts enriched in flavonol glycosides [32,55,56].

The biphasic *c-myb* response observed with Nieddera at intermediate concentrations (around 250 µg/mL) may reflect a transient adaptive transcriptional response, aimed at sustaining proliferation under moderate stress conditions. However, at higher concentrations, when oxidative and mitochondrial stress exceeds a critical threshold, this compensatory mechanism appears to be impaired, resulting in *c-myb* downregulation and a concomitant decline in cell viability. Similar hormetic-like responses have been widely reported for polyphenols in cancer cells and are increasingly recognized as a hallmark of redox-active phytochemicals [10,13,57].

However, since all biological assays were performed using complex leaf extracts, the observed cellular responses cannot be unequivocally attributed to individual flavonols, anthocyanins, or other specific constituents. Rather, they likely reflect the combined activity of multiple phytochemicals, whose additive, synergistic, or antagonistic interactions cannot be excluded. Therefore, the associations between specific phenolic classes and the observed biological effects should be regarded as mechanistic hypotheses that require validation using isolated compounds, defined phytochemical combinations, or fractionated extracts.

### 3.4. Apoptosis

**Figure 5:**
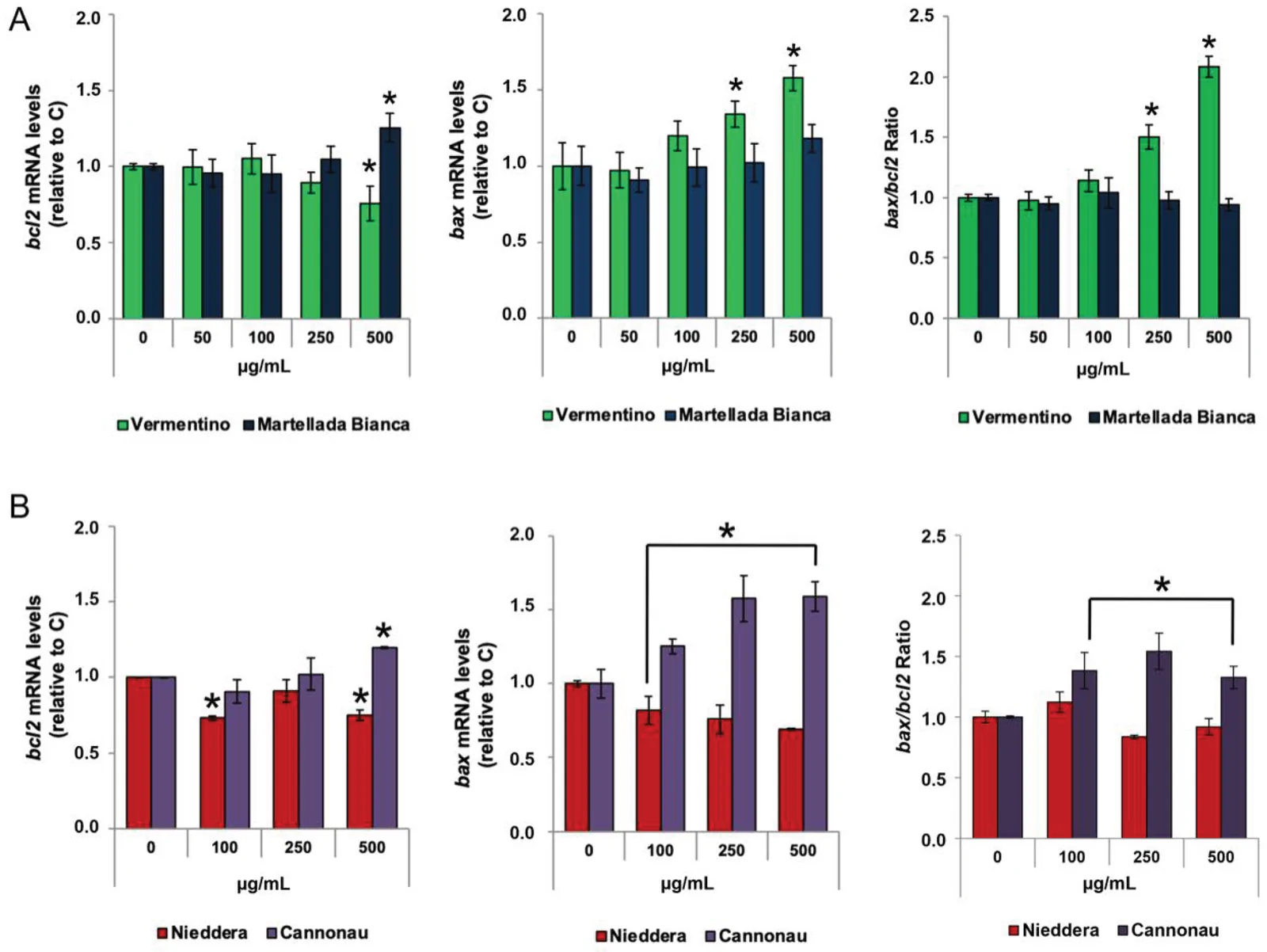
Relative expression of apoptosis-related genes *bax* and *bcl2* in RKO cells treated with Vermentino and Martellada Biaqnca (**A**) and with Nieddera and Cannonau (**B**) extracts. *bax/bcl2* ratio was also considered for each condition. Cells were exposed to increasing concentrations of extracts for 24 h, and gene expression was quantified by qRT-PCR using the 2^−ΔΔCt^ method, normalized to 18S rRNA. Data are presented as mean ± SD. Statistical significance was assessed by one-way ANOVA followed by Dunnett’s multiple comparisons test. * *p* ≤ 0.05 was considered statistically significant.

**Figure 6:**
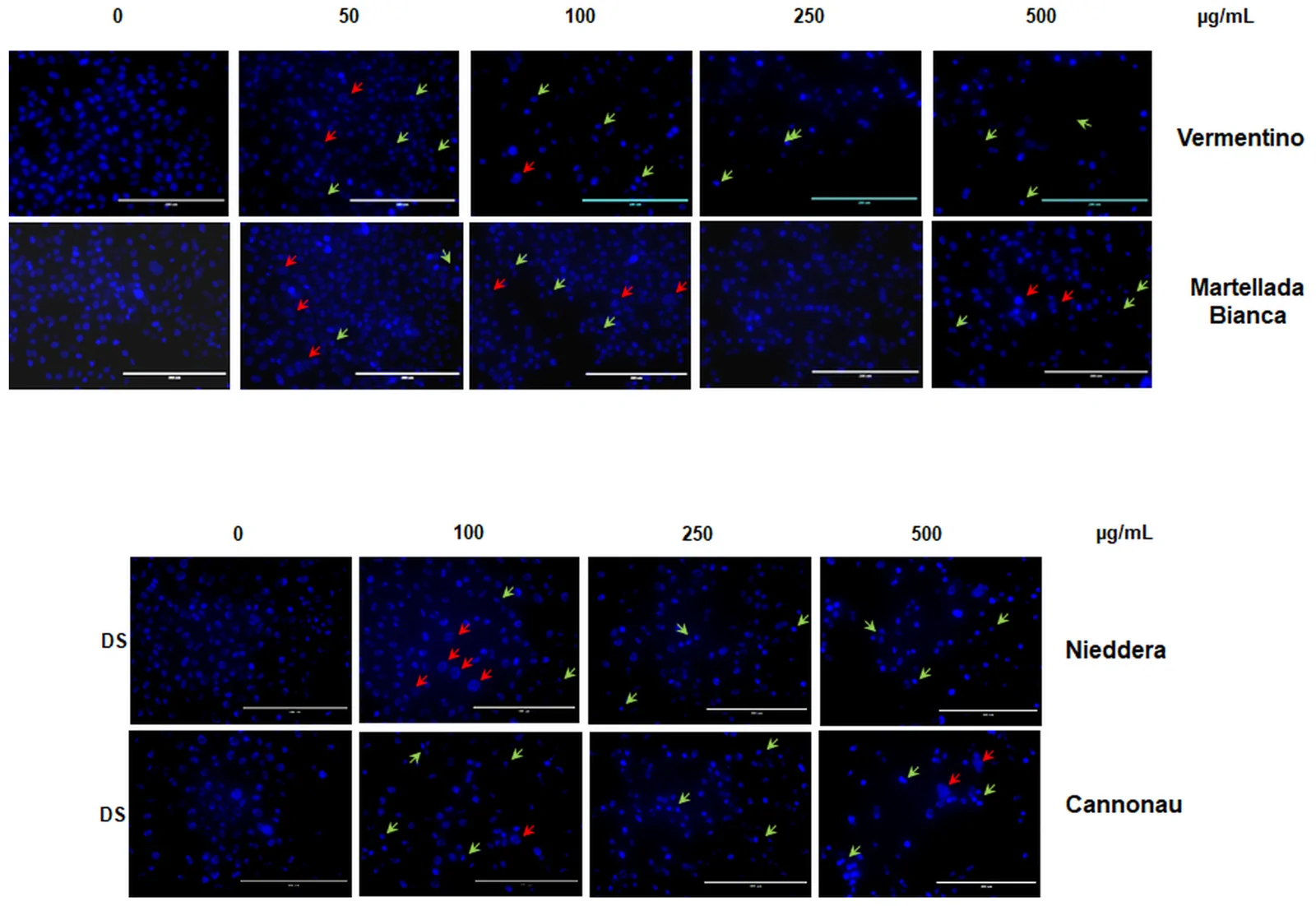
Representative fluorescence microscopy images of DAPI-stained RKO cells following treatment with *Vitis vinifera* leaf extracts. Control cells exhibit normal nuclear morphology (green arrow) with homogeneous chromatin distribution, whereas treated cells show typical apoptotic features (red arrow), including chromatin condensation, nuclear shrinkage, and fragmentation. Images are representative of three independent experiments.

To further elucidate whether the reduction in cell viability observed in RKO cells was associated with programmed cell death, the expression of the apoptosis-related genes *bax* and *bcl2* was analyzed at the mRNA level (Figure 5), together with morphological evaluation by DAPI nuclear staining (Figure 6).

As shown in Figure 5, treatment with grapevine leaf extracts promoted a dose-dependent shift toward a pro-apoptotic transcriptional profile, characterized by increased *bax* expression in RKO cells treated with Vermentino, Martellada Bianca and Cannonau extracts and reduced or unchanged BCL2, resulting in a marked increase in the *bax*/*bcl2* ratio at higher concentrations when treated with Vermentino and Nieddera extracts. This ratio is a molecular determinant of the intrinsic apoptotic pathway and reflects the balance between pro- and anti-apoptotic BCL-2 family members [36,37]. Anthocyanins have also been reported to promote mitochondrial apoptosis by increasing the *bax/bcl2* ratio and facilitating mitochondrial membrane depolarization [54]. Although the present study was not designed to dissect the contribution of individual phytochemicals, the coexistence of flavonols, anthocyanins, and other constituents may collectively contribute to the pro-apoptotic effects observed in Nieddera-treated cells. Potential additive or synergistic interactions among these compounds cannot be excluded.

The transcriptional data were corroborated by DAPI-based nuclear morphology analysis, which revealed clear apoptotic features in treated RKO cells (Figure 6).

While control cells displayed uniformly stained nuclei with homogeneous chromatin distribution, extract-treated cells exhibited chromatin condensation, nuclear shrinkage, and nuclear fragmentation, which became progressively more evident with increasing extract concentration. These morphological alterations are widely recognized hallmarks of apoptosis and are routinely used as complementary indicators of apoptotic execution in fluorescence microscopy-based assays [58,59,60].

From a mechanistic standpoint, the observed modulation of the *bax*/*bcl2* axis is consistent with the possible involvement of mitochondrial apoptotic signaling and redox-associated cellular stress, as inferred from MTT assay results and supported by the flavonol-rich chemical composition of the extracts. Flavonols such as quercetin and isorhamnetin derivatives have been shown to induce apoptosis in colorectal cancer cells through mitochondrial outer membrane permeabilization, ROS accumulation, and activation of intrinsic apoptotic signaling cascades [10,23]. In particular, isorhamnetin glycosides have been reported to trigger apoptosis in human colon cancer models by directly impairing mitochondrial integrity, in agreement with the transcriptional and morphological changes observed in RKO cells [10].

These findings are further supported by recent literature demonstrating that polyphenol-rich grape-derived matrices modulate BCL-2 family proteins and promote apoptosis preferentially in tumor cells, while exerting limited toxicity at lower concentrations [3,4,13,38]. Notably, similar bioactive properties have been reported for grapevine leaf extracts, reinforcing the concept that leaves represent a biologically relevant and underexploited source of anticancer polyphenols [5,13,38]. To further validate the involvement of apoptotic pathways, caspase-3/7 activity was subsequently investigated. The nuclear condensation and fragmentation observed after treatment are consistent with the coordinated action of multiple phenolic constituents capable of inducing mitochondrial dysfunction and apoptotic cell death [54]. In this context, anthocyanins may reinforce the pro-apoptotic activity already associated with flavonol-rich extracts.

### 3.5. Caspase-3/7 Activation Further Supports Apoptosis-Associated Responses

Caspase-3/7 activity was measured in RKO cells following treatment with increasing concentrations of grapevine leaf extracts from Vermentino, Martellada Bianca, Cannonau, and Nieddera.

**Figure 7:**
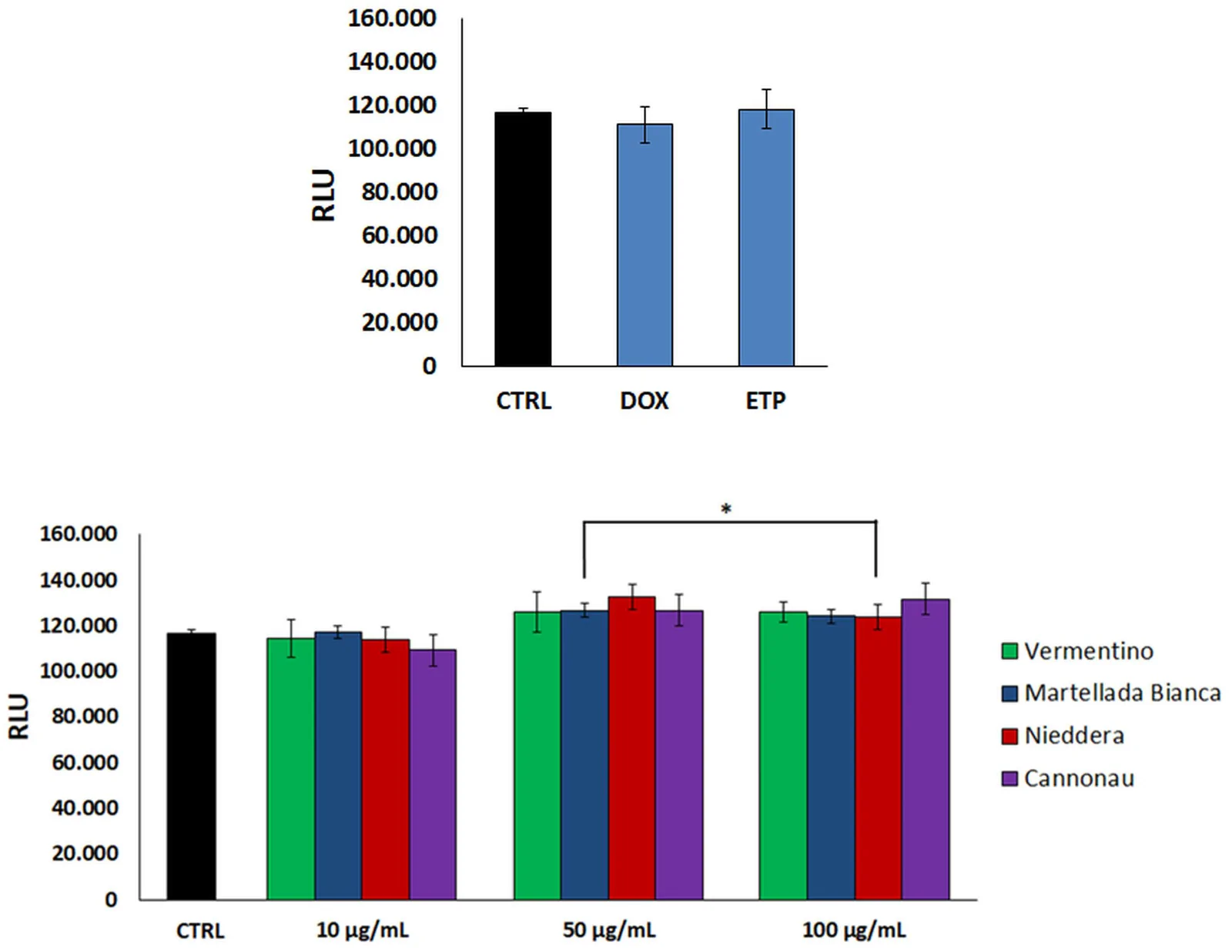
Caspase-3/7 activity in RKO cells treated with grapevine leaf extracts. Luminescence-based caspase-3/7 activity assay following treatment with Vermentino, Martellada Bianca, Cannonau, and Nieddera extracts at increasing concentrations (10–100 μg/mL). Data are expressed as mean ± SD. Statistical significance was assessed by one-way ANOVA followed by Dunnett’s multiple comparisons test. * *p* ≤ 0.05 was considered statistically significant.

As shown in Figure 7, treatment with Vermentino extract resulted in a modest but significant increase in caspase-3/7 activity at 100 μg/mL (*p* = 0.025), whereas lower concentrations did not significantly alter enzymatic activity. Similarly, Martellada Bianca induced significant caspase activation at both 50 μg/mL and 100 μg/mL (*p* = 0.023 and *p* = 0.024, respectively). Cannonau extract displayed a dose-dependent increase, reaching statistically significant activation at 50 μg/mL (*p* = 0.009) and 100 μg/mL (*p* = 0.035). In contrast, Nieddera showed significant activation only at intermediate concentrations (50 μg/mL, *p* = 0.008), suggesting a cultivar-dependent modulation of apoptotic signaling.

Caspase-3 and caspase-7 are key executioner proteases of the apoptotic cascade and represent essential mediators of the terminal phase of programmed cell death. Their activation results in the cleavage of structural and regulatory proteins, DNA fragmentation, and controlled dismantling of cellular components, hallmark features of apoptotic execution [37,58]. More recent studies have further highlighted the central role of executioner caspases as molecular regulators of programmed cell death pathways and cellular homeostasis, particularly in cancer cells where apoptosis resistance represents a hallmark of tumor progression [61,62]. Previous studies have demonstrated that cyanidin-based anthocyanins activate the intrinsic apoptotic pathway through mitochondrial cytochrome c release and caspase-3 activation. Accordingly, the anthocyanins identified in Nieddera, together with flavonols and other phytochemical constituents, may contribute to the observed modulation of caspase-3/7 activity. However, the contribution of individual compounds cannot be resolved from the present experiments, and potential interactions among extract constituents should be considered [54].

In the intrinsic apoptotic pathway, activation of caspase-3/7 generally occurs downstream of mitochondrial apoptotic signaling events, including cytochrome c release and apoptosome formation. Recent evidence indicates that dysregulation of caspase activity contributes to tumor survival and therapy resistance, reinforcing the importance of caspase-dependent apoptosis as a major therapeutic target in colorectal cancer [51,63].

From a mechanistic perspective, activation of caspase-3/7 is closely associated with oxidative stress-mediated apoptosis. ROS accumulation can trigger mitochondrial membrane permeabilization and promote activation of executioner caspases, including caspase-3 and caspase-7, thereby linking redox imbalance to apoptotic signaling cascades [25,26,64]. Furthermore, according to recent studies investigating natural bioactive compounds, ROS-dependent activation of caspase signaling has been proposed as a common mechanism through which phytochemicals induce apoptosis in colorectal cancer models [53,65,66].

Although caspase activation observed in the present study was moderate in magnitude, its occurrence at intermediate and higher extract concentrations supports the involvement of regulated apoptotic processes rather than immediate necrotic damage. This progressive activation pattern is consistent with current models of apoptosis induced by redox-active phytochemicals, in which mitochondrial stress precedes full activation of executioner caspases and cellular dismantling [10,24].

Overall, the integration of caspase-3/7 activity with transcriptional modulation of bax/bcl2 and morphological evidence of nuclear fragmentation supports the hypothesis that grapevine leaf extracts activate intrinsic mitochondrial apoptosis in colorectal cancer cells through redox-dependent mechanisms.

### 3.6. Gastrointestinal Fate, Metabolism, and Local Bioavailability of Grapevine Leaf Flavonols

Although the in vitro experiments suggest biological activity of grapevine leaf extracts in RKO colorectal cancer cells, the physiological relevance of these findings depends on the gastrointestinal stability and intestinal bioavailability of the identified flavonols. LC–HRMS analysis revealed that the extracts are mainly composed of glycosylated derivatives of quercetin, kaempferol, and isorhamnetin, chemical forms that are only partially absorbed in the small intestine and extensively metabolized after ingestion [28,67].

During digestion, flavonol glycosides remain relatively stable under gastric conditions but undergo hydrolysis at the intestinal brush border by lactase-phlorizin hydrolase and cytosolic β-glucosidases, releasing aglycones that are rapidly conjugated within enterocytes through glucuronidation, sulfation, and methylation. These metabolic transformations strongly influence the bioavailability and biological activity of flavonols and their circulating metabolites [67,68]. Consequently, circulating forms consist mainly of conjugated metabolites, while a significant proportion escapes absorption and reaches the colon [29,31].

This aspect is particularly relevant in the context of colorectal cancer. In the colon, gut microbiota catalyzes extensive biotransformation of flavonoids into low-molecular-weight phenolic acids that retain biological activity and directly interact with colonocytes [30,69]. Microbial metabolism of dietary flavonoids has been strongly associated with the modulation of proliferation, apoptosis, and inflammatory pathways involved in colorectal carcinogenesis [70].

Interestingly, O-methylated flavonols such as isorhamnetin derivatives, abundant in Cannonau and Nieddera extracts, exhibit increased metabolic stability and membrane permeability compared with non-methylated flavonols, potentially prolonging intestinal exposure [28]. Therefore, qualitative differences in flavonols composition among cultivars may directly influence local bioactivity within the colorectal environment.

Taken together, these considerations suggest that grapevine leaf flavonols are likely to act primarily at the intestinal level rather than systemically, supporting the physiological relevance of the observed modulation of proliferation and apoptosis in RKO cells and reinforcing their potential role in colorectal cancer prevention strategies [30,70].

This local bioavailability scenario further supports the use of colorectal cellular models as a physiologically relevant system to investigate the biological activity of grapevine leaf polyphenols.

## 4. Conclusions

The present study suggests that hydroalcoholic extracts obtained from Sardinian *Vitis vinifera* leaves possess cultivar-specific phenolic profiles characterized predominantly by flavonol glycosides, together with a distinct anthocyanin fraction in the red cultivar Nieddera. LC–HRMS analysis identified glycosylated derivatives of quercetin, kaempferol, and isorhamnetin as the major phenolic constituents, while targeted analysis further revealed the presence of cyanidin-3-O-glucoside and peonidin-3-O-glucoside in Nieddera, expanding the phytochemical characterization of these underexploited grapevine by-products. The coexistence of multiple phenolic subclasses highlights the metabolic diversity of Sardinian autochthonous cultivars and reinforces their potential as valuable sources of bioactive compounds.

Electrochemical characterization by cyclic voltammetry revealed marked cultivar-dependent differences in redox behavior, with Vermentino and Martellada Bianca displaying the highest electrochemical activity. When integrated with the phytochemical analysis, these findings indicate that the electrochemical fingerprint of each extract reflects the combined contribution of different phenolic subclasses rather than the abundance of a single compound family, supporting the relevance of electrochemical approaches for the functional characterization of complex plant matrices.

At the biological level, grapevine leaf extracts induced a dose-dependent reduction in colorectal cancer cell metabolic activity accompanied by modulation of oxidative stress, antioxidant defense, proliferation-related pathways, and apoptosis. The coordinated regulation of ROS production, SOD2/CAT balance, c-MYB and c-MYC expression, together with the increased BAX/BCL2 ratio, caspase-3/7 activation, and the morphological evidence of nuclear condensation and fragmentation, supports a mechanism involving redox-mediated mitochondrial apoptosis. Given the complexity of the extracts, the observed biological responses cannot be assigned to individual phenolic compounds or subclasses. Flavonol glycosides, which represent the predominant phenolic constituents, together with anthocyanins and other phytochemicals, may collectively contribute to the modulation of intracellular redox homeostasis and apoptosis-related signaling. Potential additive, synergistic, or antagonistic interactions among these constituents cannot be excluded and warrant further investigation using isolated compounds, defined combinations, and fractionated extracts.

Overall, this work provides an integrated characterization linking cultivar-specific phytochemical signatures with electrochemical properties and biological responses in colorectal cancer cells. These findings support the concept that the biological activity of grapevine leaf extracts arises from the synergistic interaction among different phenolic classes rather than from individual compounds alone.

Beyond their biological potential, the present findings contribute to the sustainable valorization of Sardinian viticultural biodiversity by demonstrating that grapevine leaves, traditionally considered an agricultural by-product, represent a valuable reservoir of cultivar-specific phenolic compounds suitable for high-value applications within a circular bioeconomy framework. Further studies incorporating non-tumoral intestinal models, advanced intestinal co-culture systems, bioavailability and metabolism assessments, and *in vivo* investigations will be necessary to establish the physiological relevance, safety, and selectivity of these extracts and to further evaluate their translational potential.

## Figures and Tables

**Table 1:** Operating parameters of ASE procedure.

Temperature (°C)	25
Pressure (psi)	1500
Number of cycles	4
Static extraction time (min)	5
Flush volume (mL)	12
Purge time (min)	1
Extraction solvent (%)	Ethanol/water (40/60 *v*/*v*)
Water	Ultrapure

**Table 2:** Quantification of flavonol compounds (mg g^−1^ ± SD) in hydroalcoholic extracts obtained from *Vitis vinifera* leaves by LC-HRMS analysis.

Compound	Martellada Bianca	Nieddera	Cannonau
Myricetin 3-O glucoside	0.06 ± 0.02	0.12 ± 0.01	0.43 ± 0.03
Eridictyol 7-glucoside	0.11 ± 0.03	0.75 ± 0.03	0.41 ± 0.01
Quercetin 3-O rutinoside	0.06 ± 0.05	0.14 ± 0.04	0.17 ± 0.01
Quercetin 3-O galactoside	0.57 ± 0.02	0.94 ± 0.03	0.40 ± 0.03
Quercetin 3-O glucoside	2.92 ± 0.04	5.17 ± 0.05	2.12 ± 0.05
Quercetin 3-O-(6 acetyl) glucoside	0.14 ± 0.01	–	–
Kaempferol 3-O galactoside	0.39 ± 0.03	0.44 ± 0.01	–
Kaempferol 3-O rutinoside	0.08 ± 0.04	0.08 ± 0.01	–
Kaempferol 3-O glucoside	1.97 ± 0.01	1.35 ± 0.02	0.18 ± 0.02
Isorhamnetin glucoside	6.14 ± 0.06	14.67 ± 0.03	22.32 ± 0.07
Total flavonols	12.43	23.67	26.03

**Table 3:** Quantification of anthocyanin compounds (mg g^−1^ ± SD) in hydroalcoholic extracts obtained from *Vitis vinifera* leaves by LC-HRMS analysis.

Compound	Nieddera	Cannonau
Cyanidin-3,5-*O*-Diglucoside	0.29 ± 0.02	–
Delphinidin-3-*O*-Glucoside	–	–
Cyanidin-3-*O*-Glucoside	3.25 ± 0.07	0.29 ± 0.03
Petunidin-3-*O*-Glucoside	0.07 ± 0.01	–
Peonidin-3-*O*-Glucoside	2.07 ± 0.04	0.13 ± 0.01
Petunidin-3-*O*-Caffeoylglucoside	0.36 ± 0.01	–
Malvidin-3-*O*-Glucoside	–	–
Delphinidin-3-*O*-*p*-coumaroylglucoside	0.04 ± 0.00	–
Cyanidin-3-*O*-*p*-coumaroylglucoside	–	–
Malvidin-3-*O*-*p*-coumaroylglucoside	0.05 ± 0.00	–
Peonidin-3-*O*-*p*-coumaroylglucoside	0.03 ± 0.00	–
Total anthocyanin	6.16	0.42

## Data Availability

The data presented in this study are available on request from the corresponding author. The data are not publicly available due to privacy and ethic.

## Supplementary Materials

The following supporting information can be downloaded at https://www.mdpi.com/article/10.3390/antiox15091153/s1, Figure S1. Chemical characterization of polyphenols in leaf extracts of Vermentino (A), Nieddera (B), Cannonau (C) and Martellada Bianca (D) by LC-MS/DAD Analysis; Figure S2. Chemical characterization of anthocyanins in leaf extracts from Vermentino (A), Nieddera (B), Cannonau (C), and Martellada Bianca (D) by LC-MS/DAD analysis.
