# Supplementary material for: Valorization of Sardinian Grapevine Leaves as a Sustainable Source of Flavonols with Anticancer Potential
Source: Antioxidants (Basel). 2026 Sep 10;15(9):1153. doi: 10.3390/antiox15091153 (PMC13603752; doi:10.3390/antiox15091153)
Supplement: Supplementary file 1 [file antioxidants-15-01153-s001.zip › antioxidants-4483133-supplementary.pdf]

VALORIZATION OF SARDINIAN GRAPEVINE LEAVES AS A SUSTAINABLE SOURCE OF FLAVONOLS WITH ANTICANCER POTENTIAL

Ylenia Spissu, Maria Lauda Tomasi, Carla Cossu, Andrea Floris, Emanuela Azara, Gavina Serra, Irene Marchesi, Francesco Paolo Fiorentino, Gaia Rocchitta, Riccarda Zappino and Antonio Barberis

Supplementary materials

Figure S1. Chemical characterization of polyphenols in leaf extracts of Vermentino (A), Nieddera (B), Cannonau (C) and Martellada Bianca (D) by LC-MS/DAD Analysis

Figure S2. Chemical characterization of anthocyanins in leaf extracts from Vermentino (A), Nieddera (B), Cannonau (C), and Martellada Bianca (D) by LC-MS/DAD analysis.

Figure S1. Chemical characterization of polyphenols in leaf extracts of Vermentino (A), Nieddera (B), Cannonau (C) and Martellada Bianca (D) by LC-MS/DAD Analysis.

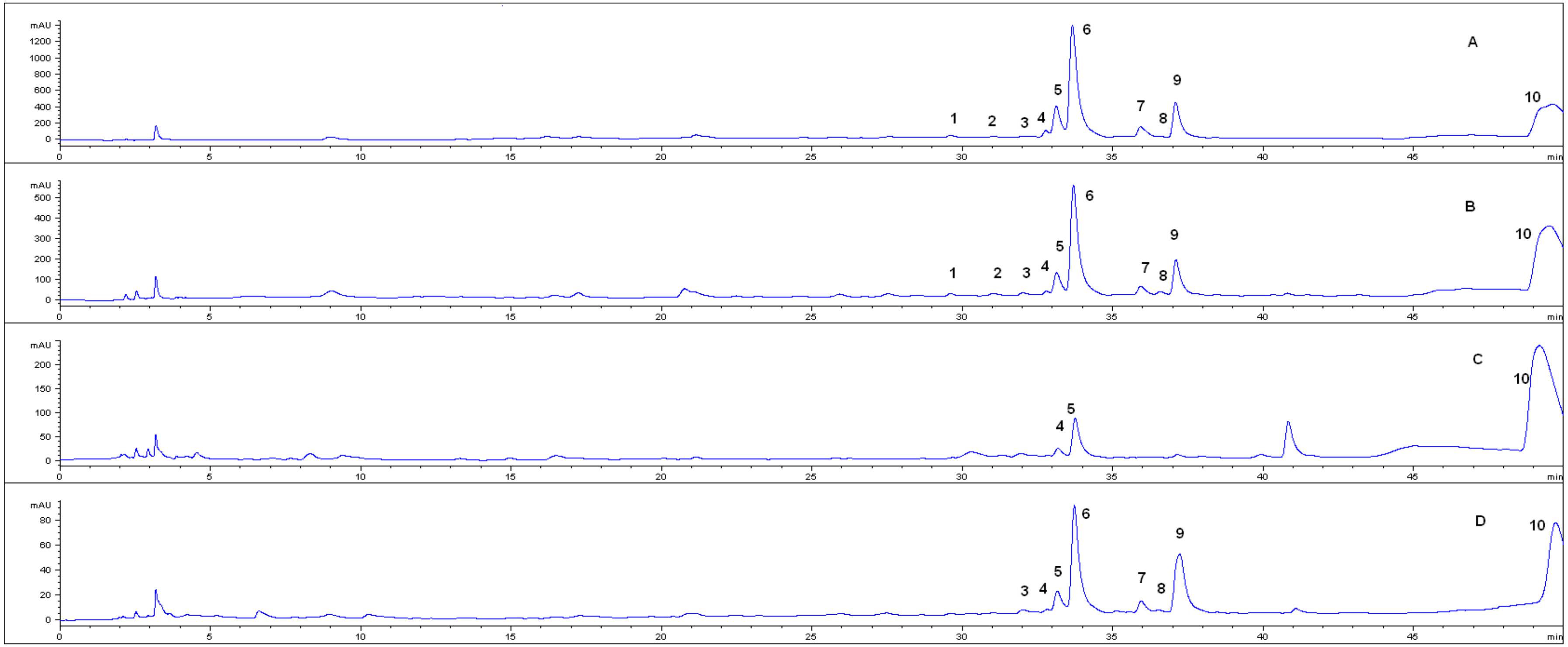

1 = Myricetin 3-O glucoside; 2 = Eridictyol 7-glucoside; 3 = Quercetin 3-O rutinoside; 4 = Quercetin 3-O galactoside; 5 = Quercetin 3-O glucoside; 6 = Kaempferol 3-O galactoside; 7 = Kaempferol 3-O rutinoside; 8 = Kaempferol 3-O glucoside; 9 = Quercetin 3-O-(6 acetyl)glucoside; 10 = Isorhamnetin glucoside.

Figure S2. Chemical characterization of anthocyanins in leaf extracts from Vermentino (A), Nieddera (B), Cannonau (C), and Martellada Bianca (D) by LC-MS/DAD analysis.

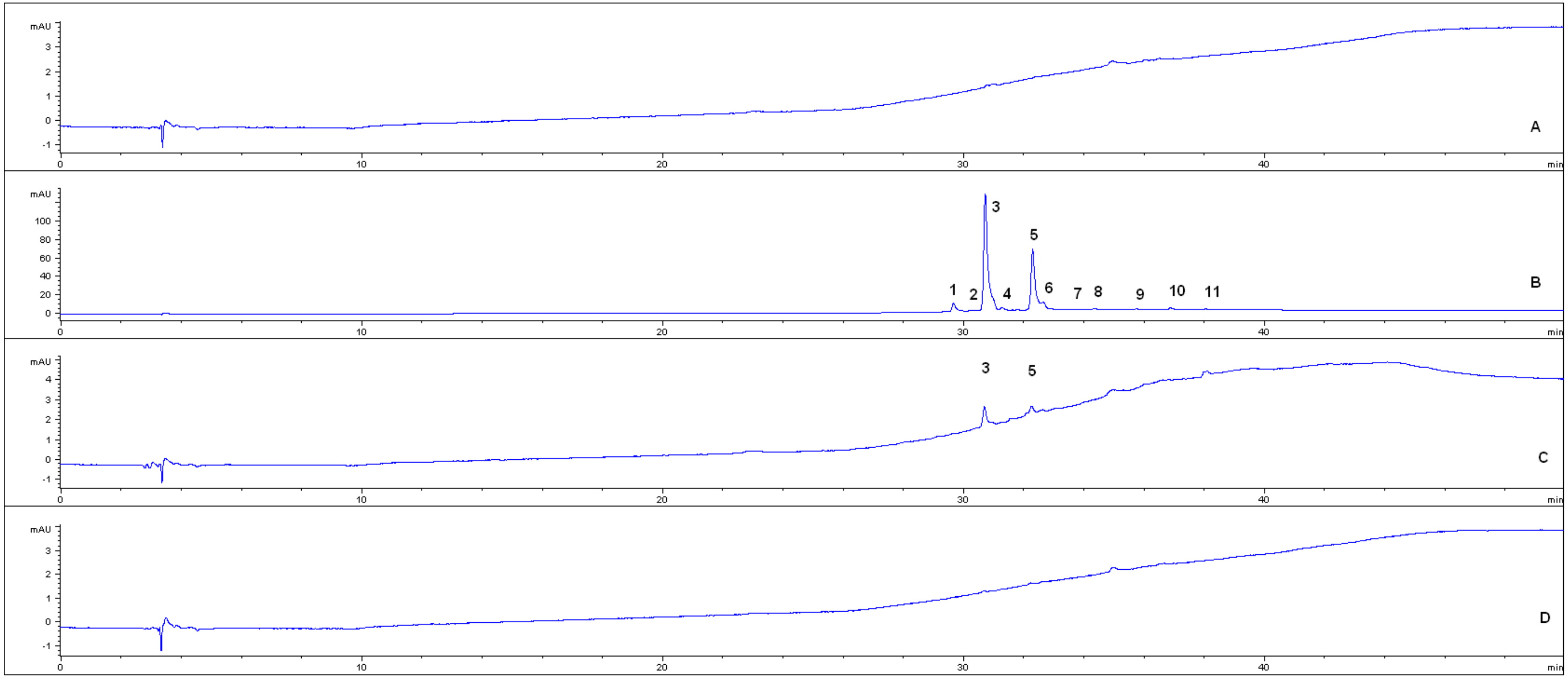

1 = Cyanidin-3,5-*O*-Diglucoside; 2 = Delphinidin-3-*O*-Glucoside; 3 = Cyanidin-3-*O*-Glucoside; 4 = Petunidin-3-*O*-Glucoside; 5 = Peonidin-3-*O*-Glucoside; 6 = Petunidin-3-*O*-Caffeoylglucoside; 7 = Malvidin-3-*O*-Glucoside; 8 = Delphinidin-3-*O*-*p*-coumaroylglucoside; 9 = Cyanidin-3-*O*-*p*-coumaroylglucoside; 10 = Malvidin-3-*O*-*p*-coumaroylglucoside; 11 = Peonidin-3-*O*-*p*-coumaroylglucoside
